# A mixed-methods exploration of cognitive dispositions to respond and clinical reasoning errors with multiple choice questions

**DOI:** 10.1186/s12909-018-1372-2

**Published:** 2018-11-23

**Authors:** Luke T. Surry, Dario Torre, Robert L. Trowbridge, Steven J. Durning

**Affiliations:** 1 Department of Medicine, Uniformed Services University of the Health Sciences - F. Hébert School of Medicine, Bethesda, MD 20814 USA; 2 Department of Medicine, Tufts University School of Medicine, Boston, MA USA; 3 Maine Medical Centre, Portland, ME USA

**Keywords:** Clinical reasoning, Cognitive disposition to respond (CDR), Reasoning errors, Medical errors, Medical decision making

## Abstract

**Background:**

Cognitive dispositions to respond (i.e., cognitive biases and heuristics) are well-established clinical reasoning phenomena. While thought by many to be error-prone, some scholars contest that these cognitive dispositions to respond are pragmatic solutions for reasoning through clinical complexity that are associated with errors largely due to hindsight bias and flawed experimental design. The purpose of this study was to prospectively identify cognitive dispositions to respond occurring during clinical reasoning to determine whether they are actually associated with increased odds of an incorrect answer (i.e., error).

**Methods:**

Using the cognitive disposition to respond framework, this mixed-methods study applied a constant comparative qualitative thematic analysis to transcripts of think alouds performed during completion of clinical-vignette multiple-choice questions. The number and type of cognitive dispositions to respond associated with both correct and incorrect answers were identified. Participants included medical students, residents, and attending physicians recruited using maximum variation strategies. Data were analyzed using generalized estimating equations binary logistic model for repeated, within-subjects measures.

**Results:**

Among 14 participants, there were 3 cognitive disposition to respond categories – *Cognitive Bias*, *Flaws in Conceptual Understanding*, and *Other Vulnerabilities* – with 13 themes identified from the think aloud transcripts. The odds of error increased to a statistically significant degree with a greater per-item number of distinct *Cognitive Bias* themes (OR = 1.729, 95% CI [1.226, 2.437], *p* = 0.002) and *Other Vulnerabilities* themes (OR = 2.014, 95% CI [1.280, 2.941], *p* < 0.001), but not with *Flaws in Conceptual Understanding* themes (OR = 1.617, 95% CI [0.961, 2.720], *p* = 0.070).

**Conclusion:**

This study supports the theoretical understanding of cognitive dispositions to respond as phenomena associated with errors in a new prospective manner. With further research, these findings may inform teaching, learning, and assessment of clinical reasoning toward a reduction in patient harm due to clinical reasoning errors.

**Electronic supplementary material:**

The online version of this article (10.1186/s12909-018-1372-2) contains supplementary material, which is available to authorized users.

## Background

Nearly 20 years ago, *To Err is Human* called the national consciousness to the tragedy of error in medical care [1]. Recent studies place medical error as the 3rd leading cause of death in the United States – behind only Heart Disease and Cancer [2]. Diagnostic error, a major sub-type of medical error, accounts for approximately 10% of patient deaths and between 6 and 17% of adverse events in the hospital per autopsy and chart review studies, respectively [3]. It is estimated to occur, on average, in 15% of cases completed by physicians in clinical specialties (e.g., Family Medicine, Internal Medicine, Emergency Medicine, etc.) [4, 5].

Despite the tremendous personal and public health burdens of diagnostic error, there has been relative inattention directed towards understanding and reducing it [3]. This may be due to a number of factors inherent to diagnostic errors, including difficulty in defining and identifying them, their subjective nature, delays in recognizing them, their complex and multifactorial causation [6, 7], and the lack of clear solutions [8]. Also, the typical indicator that error occurred – patient harm – may not always be detected [3]. In addition, the current healthcare delivery system cultivates “a culture that discourages transparency and disclosure of diagnostic errors—impeding attempts to learn from these events and improve diagnosis,” [3] preventing clinicians and institutions from receiving the feedback from real-world clinical practice necessary to improve diagnostic reliability [9].

Beyond these obstacles, diagnosis is complex. The general model of diagnosis from *Improving Diagnosis* (2015) describes this complexity as the interaction of several dynamic processes (e.g., health system, information sharing, communication, etc.) and participants (e.g., patient, clinician, laboratory technician, radiologist, etc.) over time, all interacting with the processes of clinical reasoning [3]. Several current views of clinical reasoning, which can be defined as the steps up to and including establishing a diagnosis and/or therapy, suggest the complexity of this process is further compounded by the influence of several other contextual factors (e.g., fatigue, emotion, stress, cognitive load, etc.) that occur with making clinical decisions [10–15]. Clinical reasoning is also thought to be influenced by several specific internal cognitive vulnerabilities, “especially those associated with failures in perception, failed heuristics, and biases collectively, referred to as cognitive dispositions to respond (CDRs)” [8].

The association of these contextual factors and CDRs (i.e., cognitive biases and heuristics) with diagnostic errors has been previously described by Kahneman and Tversky with additional contributions by Croskerry and others [3, 8, 14, 16–20]. While the existence of such biases and heuristics are well-established as system 1 (automatic) processes that are distinct from system 2 (analytic) processes in the dual process theory framework [6, 16–20], the error-prone nature of CDRs with diagnostic error remains controversial [21, 22]. In part, this controversy is because “[e]mpirical evidence on the cognitive mechanisms underlying such flaws and effectiveness of strategies to counteract them is scarce” [23]. In addition, research on diagnostic errors is retrospective in nature and plagued by ambiguity and variation in defining and detecting reasoning errors [3]. Moreover, hindsight bias may increase the detection of heuristics or biases when researchers are cued by the presence of an error [7]. Furthermore, there is continued debate as to whether CDRs might actually contribute as pragmatic strengths to diagnostic accuracy [21, 22, 24], instead of being vulnerabilities associated with error [25].

In sum, the empiric support for the dual process theory-based understanding of CDRs as associated with an increased likelihood of diagnostic errors is limited. To better fill this gap in our understanding, more robust means of detecting error in clinical practice [5] and novel experimental approaches are necessary. We believe using multiple-choice questions (MCQs), widely applied in standardized exams to assess clinical reasoning and found to elicit real-world reasoning processes in previous research [26–28], supplemented with a think aloud (TA) protocol can provide valuable insight into such errors. Furthermore, MCQs hold the advantage of having an a priori distinct correct answer, allowing for a clear, prospective analysis that limits hindsight bias.

In this mixed-methods study, we explore what CDRs, if any, are present when medical students, residents, and attending physicians solve MCQs and how these CDRs may relate to incorrect answer selection (i.e., error). We hypothesized that CDRs detected in think alouds completed during answering high-quality clinical-vignette MCQs, a task previously shown to elicit clinical reasoning processes [26–28], would be associated with errors. Such a finding would be consistent with views of dual process theory posited by Croskerry, Kahneman, and Tversky and support the position that system 1 (automatic) reasoning processes like CDRs may contribute to error [8, 14, 16, 20]. In addition, such findings would further support for the assessment and study of clinical reasoning using think aloud supplemented MCQs.

## Methods

### Participants

From May to November 2016, we used a maximum variation recruiting approach through a series of recruiting emails sent to list-serves for medical students, Internal Medicine (IM) residents, and IM-trained attending physicians at a single institution. We targeted this heterogeneous sample to more fully study the phenomenon of CDRs in clinical reasoning across the spectrum of individuals who participate in clinical reasoning processes.

### Design

We combined real-time rich data collection of thought processes using a well-established think aloud (TA) approach with outcomes discretely identifiable as either correct or incorrect (error) based on clinical scenarios presented in MCQs. We selected high quality, Internal Medicine clinical-vignette MCQ items with extensive psychometric data from the American College of Physicians (ACP) Medical Knowledge Self-Assessment Program (MKSAP) 15, published in 2009, and MKSAP for Students 4, published in 2008, question banks [29, 30]. Using older MKSAP questions limited potential familiarity of MCQs among participants. MKSAP and MKSAP for students were chosen as their questions undergo extensive peer review, are generally of high quality, and target medical students and faculty with different levels of difficulty.

Each participant completed the same 15 paper-based MCQ items divided over three distinct 5-item blocks (see Additional file 1, Item Selection). Consistent with the American Board of Internal Medicine (ABIM) Certification Exam, participants were allotted 2 min per item. Immediately after completing the first MCQ block, the participant was instructed to describe, in as much detail as possible, their thoughts in solving each MCQ item. This TA protocol is a well-established and commonly used approach to record cognitive processes [31].

The similarity of this immediate retrospective TA protocol to the more traditional concurrent TA is supported by precedent [28] and neuroimaging [32]. Prior to beginning this TA, participants were given an opportunity to practice with a non-medical problem; however, no prompting or questioning occurred once the TA protocol commenced. This process was repeated for each of the two remaining question blocks. To control for fatigue and priming effects, the sequence of question blocks was randomized for each participant.

LTS ran the protocol with all participants including timing the MCQ blocks, recording TAs, and collecting all other data. We transcribed audio recordings of the TAs verbatim using F5 Transcription Pro (version 3.2) software [33].

### Data analysis

We used cognitive dispositions to respond (CDRs) [8, 16] as the sensitizing conceptual framework for our qualitative thematic analysis. Consistent with the Constant-Comparative Approach (CCA), we developed our coding structure through a detailed immersion in the data with identification of the phenomena of interest, categorization of these phenomena (i.e., applying codes), and performing within- and between-item comparisons of these codes [34]. As described in the application of CCA outside of Grounded Theory, our qualitative analysis consisted of an iterative process of independent coding, group discussion, and code revision ultimately identifying a consensus framework of main categories and themes representing the data while maintaining grounding in our sensitizing framework [35]. Throughout this process, the coding framework was reviewed and revised as a group (LTS, SJD, DT). Once a consensus thematic framework for CDRs was finalized, all transcripts (*N* = 210) were coded as a group with complete agreement. Two of the three coders (SJD and DT) were blinded to the identity, experience level, and scored performance of all participants. All three coders are practicing physicians facilitating coding of utterances for evidence of System 1 processes. All coding was completed using Dedoose (version 7.5.14) qualitative data analysis software [36].

To determine if CDRs were associated with error, we completed a univariate Generalized Estimating Equations (GEE) multiple logistic regression model for repeated within-subjects measurements to account for 15 items completed by each of 14 participants. The binary dependent variable was the MCQ answer (reference group – correct answer; event group – incorrect answer). Independent variables (i.e., predictors) included training status (trainee vs. attending), where trainee was defined as medical student or resident, and the per-item number of coded CDR themes in each of the 3 identified CDR categories. Hybrid, Type III analysis was completed for main effects parameter estimates with 95% confidence intervals. All statistical analyses were completed using Microsoft Excel 15.3 [37] and IBM SPSS Statistics Version 22 [38].

## Results

Fourteen participants completed the protocol of 15 MCQ-items for a total of 210 items. Overall, 146 (69.5%) MCQ items were answered correctly by participants in this study, compared to expected performance of approximately 64% correct based on MKSAP data (see Additional file 1). Sixty-four (30.5%) items were scored as incorrect, one of which had no answer selected. Participants included 3 medical students, 5 IM residents, and 6 attendings. Residents included 2 post-graduate year (PGY) 1 trainees as well as 2 trainees in PGY2 and 1 trainee in PGY3. The average age was 35.6 years (range = 24–69). In total, 58,760 words (i.e., 205 pages) from TA transcripts were included in the analysis. The datasets generated during and/or analyzed during the current study are available from the corresponding author on reasonable request.

### Categories and themes

We identified 13 distinct themes in our data that fell into 3 categories (see Table 1 for categories, themes and definition of biases). The category of *Cognitive Biases* included themes of Anchoring Bias, Availability and Non-availability Bias, Commission Bias, Gambler’s Fallacy, Omission Bias, Premature Closure, Playing the Odds, and Representativeness Restraint. The category of *Flaws in Conceptual Understanding* included themes of Perceptual Flaws, Inappropriate Rule Application, and Incomplete Conceptual Knowledge Structure. Finally, the category of “*Other” Vulnerabilities* included themes of Marked Uncertainty and Emotional Reactions. All themes were well represented in the data, but 6 themes - Gambler’s Fallacy, Playing the Odds, Premature Closure, Commission Bias, Omission Bias, and Perceptual Flaws - were noted in 10% or less of all items (see Table 2 for theme frequency). At least one CDR was coded in 162 of 210 total items (77%), including all 64 items answered incorrectly and 98 of the 146 (67%) items answered correctly. In 48 of the 146 (33%) items answered correctly, no CDRs were noted. We reached complete consensus on coding structure and the application of codes. All transcripts were coded. Saturation, assessed by a post-hoc review of all items, was achieved with no new themes emerging after the second participant to complete the protocol chronologically.

**Table 1:** Cognitive dispositions to respond (CDR) themes and representative excepts

	Representative Excerpt 1	Representative Excerpt 2
**Cognitive Biases -“representations that are systematically distorted compared to some aspect of objective reality.”** [39]		
*Anchoring Bias* - the tendency to perceptually lock onto salient features in the patient’s initial presentation too early in the diagnostic process, and failing to adjust this initial impression in the light of later information [8].	Based on the fact that there’s some sort of link to the football game, I went ahead and just went with inhalation anthrax ...umm... ‘cuz maybe the…it was disseminated ...umm... somewhere where they were sitting and…they…they all inhaled the…the …uhh… pathogen and got sick. - Participant #11, Item ID.1	I was a little bit rushed through this one, but … umm… was interested in the last couple sentences where you were looking at the patchy right lower lobe infiltrate…umm… And I…was thinking about inhalational or aspiration pneumonia… - Participant #1, Item ID.5
*Availability & Non-Availability Bias* - the disposition to judge things as being more (or less) likely, or frequently occurring, based on how readily (or not) they come to mind. Thus, recent exposure to / experience with a disease (OR medication, approach to management, etc.) may inflate the likelihood of its being diagnosed (OR used) [8].	And I know a lot of the things I’ve seen…like…kind of if you’re doing antibiotics, you always cover for pseudomonas. I remember people always saying cover for pseudomonas if we’re gonna cover for anything, so that kind of pops in my head. – Participant #9, Item ID.5	Actually what I would probably done is look up what the current therapy is, ‘cuz I haven’t treated Paget’s disease in probably 10 years, so… Umm…. uhh… I gave an answer to this question…alendronate, because it…it’s what I would have done in the past, but I actually feel inadequate about that answer. – Participant #6, Item DB.2
*Commission Bias* - results from the obligation toward beneficence, in that harm to the patient can only be prevented by active intervention. It is the tendency toward action rather than inaction [8]. *MORE IS BETTER*	I didn’t…umm… choose C or D, and if you’re going to give them a supplement of calcium, you might as well just slam them with the Vitamin D supplement as well…umm…So that’s why I chose B. I figured that would be better than just calcium alone. – Participant #1, Item DB.5	Basically it got me down to quest…to…this is test taking…this…that got me to C or to D. And then the issue was…umm…the only difference between C and D is whether or not you start a bisphosphonate …umm… at this point, or not. And…I actually wasn’t sure, but I had…I, actually, had leaned…umm… toward doing it, so I answered D. The…everything about the question says this is…this is a patient who’s likely to be on …umm… long-term prednisone, and therefore, at…umm… at risk for developing osteoporosis. And that’s it. – Participant #5, Item DB.5
*Gambler’s Fallacy* - The pretest probability that a patient will have a particular diagnosis might be influenced by preceding but independent events. Attributed to gamblers, this fallacy is the belief that if a coin is tossed ten times and is heads each time, the 11th toss has a greater chance of being tails (even though a fair coin has no memory) [8].	Umm… calcitonin, I said for the bone turnover question, so I figured it wouldn’t apply to this one. – Participant #2, Item DB.3	I didn’t think it was Dementia with Lewy Bodies, because I was looking for motor signs and symptoms there, and I had already used that answer. – Participant #11, Item NCD.4
*Omission Bias* - the tendency toward inaction and rooted in the principle of non-maleficence [8]. *LESS IS MORE*	D says to start bisphosphonate. I don’t think you necessarily start somebody bisphosphonates …uhh… without sort of confirming that diagnosis. – Participant #10, Item DB.5	maybe, alendronate…maybe that drug would work better if it was given …uhh… as a different …umm… route and class, so I thought the substituting for intravenous zoledronate [throat clear] … for the alendronate, or choice ‘D,’ was gonna be the correct one, because of the way that it modified the therapy …umm… without …uhh… adding in something new. – Participant #2, Item DB.1
*Playing the Odds (Frequency Gambling)* - the tendency in equivocal or ambiguous presentations to opt for a benign explanation on the basis that it is significantly more likely than a serious one [16].	Uhh… again, looking at the lab values, I was trying to remember what normal was, and I was going back and forth trying to figure out if testosterone was low or normal, and… decided that it was normal at 50. ...umm... and the free T4, again, guessing if that was normal or elevated, and I couldn’t recall, so I st…interpreted as normal. –Participant #8, Item DB.4	and he only lost points on recall and ...umm... the orientation section for the date though. So…and I don’t even know the date, so I’m not concerned about that. – Participant #9, Item NCD.1
*Premature Closure* - Accepting a diagnosis before it has been fully verified, essentially limiting answers or selecting final answer early. Related to anchoring [8].	So…so (stutters), from the start, I was thinking this was community-acquired pneumonia …umm… but, the only weird thing was the friend who died yesterday, so…(stutters)it didn’t really affect…I …I couldn’t really make too much sense of that, but I went ahead and selected B for the answer for that. –Participant #3, Item ID.1	Physical exam ...uhh... she has cog wheeling. So right off the bat, cog wheeling sort of triggers me to think ...umm... Parkinsonian, or ...umm... Parkinsonian dementia or related, which would be a Lewy Body dementia ...umm... So really with that, I almost skipped down to the bottom, and I say “Well this is unlikely to be Alzheimer’s or Creutzfeldt Jakob.” The Parkinsonian fits with, sort of, the characteristic exam findings ...umm... So I would say right off the bat that this is Dementia with Lewy Bodies. And I look at the other choices just to make sure I’m not missing anything, but again, they don’t really fit in terms of stepwise for vascular, or speech and ...uhh... behavioral things for frontotemporal. –Participant #14, Item NCD.3
*Representativeness Restraint* - drives the diagnostician toward looking for prototypical manifestations of disease. Yet restraining decision-making along these pattern-recognition lines may lead to atypical variants being missed [16].	determined that this couldn’t be vascular dementia, ‘cuz it wasn’t “step-wise” –Participant #3, Item NCD.4	Staph aureus would be a consolidated chest x-ray, so that’s out. –Participant #14, Item ID.2
**Flaws in Conceptual Understanding - demonstrable evidence of an incorrect or inadequate basis in knowledge of the concepts presented in the clinical vignette or addressed by the participant**		
*Perceptual Flaws* - where key information presented in the MCQ item was missed by the participant, misunderstood or misinterpreted, or instances where participants erroneously added information that they then used in their reasoning.	The blood smear showed gram negative coccobacilli…and I took…I didn’t actually see the first time, there was this blood smear. I just know that somewhere (laughing), this was found. (incomprehensible) It doesn’t really sway me either way, but what…either way he’s got some gram negative cocco….coccobacilli. – Participant #9, Item ID.4	Uhh… oh! wait a minute. She’s on pentamidine. I didn’t even notice that. Hmm… (long pause…lip smack…vocalizations)… well I don’t know the effectiveness of pentamidine in preventing pneumocystis, which is why it is there; however, as seeing that she was on the pentamidine, I should probably choose something else, although I’m not allowed to change my answer now. I would probably change it to something else. –Participant #6, Item ID.2
*Inappropriate Rule Application* - instances where participants used a general rule that was either conceptually invalid, or when a general rule was clearly used inappropriately.	So this is a older gentleman …uhh… with 2 day history of fever, cough, and yellow sputum. So its a productive cough. He’s also febrile, so that tells me that he likely has a blood stream infection somewhere. – Participant #8, Item ID.5 *(Note: This is a rule in which Fever is inappropriately equated to a Blood Stream Infection/bacteremia)*	So I scan through the list real quick and… right off the bat the last two - staphylococcus and streptococcus - I think are…are …uhh.. both very unlikely because you… you would see more of the …umm… the CBC would be different because you would see neutrophils…you’d see more of …uhh… bacterial reaction …umm… --Participant #2, Item ID.2 *(Note: This is a heuristic that Bacterial Pneumonia causes leukocytosis incorrectly applied in Immunosuppressed patient)*
*Incomplete Conceptual Knowledge Structure* - Instances when the participant demonstrated clear evidence of poor conceptual understanding or a knowledge gap (i.e., self-reflective statement of knowledge deficit, expressing factually incorrect information, etc.).	Umm… so the thought then either…either should be coverage for Nocardia or for Pseudomonas. Umm… and again, this is just a knowledge gap for me. I don’t know, in people who have bronchiectasis, if…if they are particularly predisposed to one or the other of these. – Participant #5, Item ID.5	And because …umm… it has calcifications in the spleen and mediastinum, I’m thinking this thing moves around in the blood okay without being detected very easily - so I don’t think the serology is necessarily going to happen, nor the fungal blood cultures. And so I…I assume that the urinary antigen detection …uhh… would be the most …would be the best answer …umm… because I feel like a metabolic detection would be better than trying to grow a fungus from …hoping that you catch little bits of it from either the blood or serum. – Participant #2, Item ID.3
*Semantic Discompetence (Subtheme)* - using terms incorrectly or in a manner that demonstrates very poor understanding of the concept represented by the term.	Fungal blood culture, I opted against, because he didn’t appear to show any evidence of bacteremia. – Participant #8, Item ID.3	Like, I know if its mild cognitive impairment, it’s just memory loss, it’s not something that’s actually pathologic. – Participant #9, Item NCD.1
**“Other” Vulnerabilities - possible vulnerabilities related to the CDR framework but that did not fall clearly into the other categories more clearly framed by the existing literature on CDRs.**		
*Emotional Reactions* - The influence of affective sources. Coded with the presence of verbalized affective / emotional response. Difficult to code in more detail as suggested by literature given the nature of the TA data. Only able to code fairly explicit expressions of emotion.	What confirms the diagnosis? is the question. So I know I’m not looking at diagnosis question. It seems like a second order thing, so its going to be a bit more annoying. – Participant #9, Item ID.3	Uhh…wow that is pretty close to my age…is …uhh… She’s losing memory …uhh… and worsening over the past year, which is concerning. – Participant #10, Item NCD.2
*Marked Uncertainty* - the act of selecting an answer without evidence of reasoning to support that answer. This code is often associate with the use of phrases like “just a guess” or “50/50” indicated “Guessing” OR expressions of uncertainty.	and between Salmonella and Yersinia…umm… I was not sure which one looked like safety pins…umm… I think I vaguely remember its salmonella…umm… but that was more of a 50/50 shot, but I chose salmonella for the gram negative coccobaccilli that looked like safety pins, −-Participant #1, Item ID.4	so then, I’ve got two choices…umm… that it came down to, and again, I don’t…I don’t know the intravenous bisphosphonates well enough, so I essentially just basically took a guess then and said C. – Participant #5, Item DB.3

**Table 2:** Cognitive dispositions to respond counts

Cognitive Disposition to Respond (CDR)	Answer Choice	
	Incorrect (n = 64; 30.5%)	Correct (n = 146; 69.5%)
*Cognitive Biases* (*n* = 124; 59%)	55 (85.9%)*	69 (47.3%)**
Anchoring Bias (*n* = 22; 10.5%)	12 (18.75%)*	10 (6.8%)**
Availability & Non-Availability Bias (*n* = 50; 23.8%)	24 (37.5%)*	26 (17.8%)**
Gambler’s Fallacy (*n* = 6; 2.9%)	2 (3.1%)*	4 (2.7%)**
Playing the Odds (*n* = 10; 4.8%)	5 (7.8%)*	5 (3.4%)**
Premature Closure (*n* = 20; 9.5%)	5 (7.8%)*	15 (10.3%)**
Commission Bias (n = 10; 4.8%)	7 (10.9%)*	3 (2%)**
Omission Bias (*n* = 17; 8.1%)	16 (25%)*	1 (0.7%)**
Representativeness Restraint (*n* = 54; 25.7%)	17 (26.6%)*	37 (25.3%)**
*Flaws in Conceptual Understanding* (*n* = 128; 61%)	60 (93.75%)*	68 (46.6%)**
Perceptual Flaws (*n* = 21; 10%)	4 (6.25%)*	17 (11.6%)**
Inappropriate Rule Application (*n* = 32; 15.2%)	17 (26.6%)*	15 (10.3%)**
Incomplete Conceptual Knowledge Structure (*n* = 115; 54.8%)	54 (84.4%)*	61 (41.8%)**
*Other Vulnerabilities* (*n* = 60; 28.6%)	30 (46.9%)*	30 (20.5%)**
Emotional Reaction (*n* = 38; 18.1%)	14 (21.9%)*	24 (16.4%)**
Marked Uncertainty (*n* = 34; 16.2%)	23 (35.9%)*	11 (7.5%)**

NOTE: Percentage of total items completed (*N* = 210), unless otherwise noted

*Percentage out of items answered incorrectly (*n* = 64)

**Percentage out of items answered correctly (*n* = 146)

#### Cognitive biases

We define *Cognitive Biases* as “representations that are systematically distorted compared to some aspect of objective reality” [39].

*“And I know a lot of the things I’ve seen…like…kind of if you’re doing antibiotics, you always cover for pseudomonas. I remember people always saying cover for pseudomonas if we’re gonna cover for anything, so that kind of pops in my head.”*

- Participant #9, Item ID.5

**[Availability & Non-availability Bias]**

We identified 8 separate themes in this *Cognitive Biases* category that were directly defined or closely related to CDRs traditionally described in the literature [8, 16] —Anchoring Bias, Availability and Non-Availability Bias, Commission Bias, Gambler’s Fallacy, Omission Bias, Playing the Odds, Premature Closure, and Representativeness Restraint (see Table 1). One or more themes from the *Cognitive Biases* category were noted in 124 (59%) items overall.

#### Flaws in conceptual understanding

*Flaws in Conceptual Understanding*, noted in 128 (61%) items overall, was defined as demonstrable evidence of an incorrect or inadequate basis in knowledge of the concepts presented in the clinical vignette or addressed by the participant. These themes fell within the general scope of CDRs, but were not captured in the specific cognitive biases commonly described as CDRs [8, 14, 16].

*“And because …umm… it has calcifications in the spleen and mediastinum, I’m thinking this thing moves around in the blood okay without being detected very easily - so I don’t think the serology is necessarily going to happen, nor the fungal blood cultures. And so, I…I assume that the urinary antigen detection …uhh… would be the most …would be the best answer …umm… because I feel like a metabolic detection would be better than trying to grow a fungus from …hoping that you catch little bits of it from either the blood or serum.”*

- Participant #2, Item ID.3

**[Incomplete Conceptual Knowledge Structure]**

This category included three distinct themes— Perceptual Flaws, Inappropriate Rule Application, Incomplete Conceptual Knowledge Structure (see Table 1). Perceptual Flaws, was applied in 21 (10%) items to describe instances where key information presented in the MCQ item was missed by the participant, misunderstood or misinterpreted. It was also used to describe instances where participants erroneously added information that they then used in their reasoning. Inappropriate Rule Application, was applied in 32 (15.2%) items for instances where participants use of a common “rule-of-thumb” was inappropriate. The third theme, Incomplete Conceptual Knowledge Structure, was applied in 115 (54.8%) items for instances when the participant demonstrated clear evidence of poor conceptual understanding and/or a knowledge gap (i.e., statement of knowledge deficit, expressing factually incorrect information, etc.).

#### “Other” vulnerabilities

The category of *“Other” Vulnerabilities* includes those themes that did not fall clearly into the other categories but represented additional vulnerabilities to error. Themes in this category were consistent with the broad definition of CDRs, but described phenomena beyond the more common biases and heuristics [8, 14, 16]. There were 2 themes– Marked Uncertainty and Emotional Reaction (see Table 1). Marked Uncertainty was defined as the act of selecting an answer without evidence of reasoning to support that answer. This code was often associated with the use of phrases like “just a guess,” or “50/50.” Marked Uncertainty was noted in 34 (16.2%) items overall. Emotional Reaction was defined as the verbalization of an affective response in the context of the item. It was noted in 38 (18.1%) items overall. At least one of these two themes were noted in 60 (28.6%) items overall.

*“Uhh…wow that is pretty close to my age…is …uhh… She’s losing memory …uhh… and worsening over the past year, which is concerning.”*

*–* Participant #10, Item NCD.2

**[Emotional Reaction]**

### Number of CDRs among correct versus incorrect items

Among the 146 items answered correctly, there was at least one of the 13 themes applied in 98 items and no themes in the remaining 48 items (*M* = 1.568; *SD* = 1.627). Among the 64 items answered incorrectly, all had at least one theme applied (*M* = 3.125; *SD* = 1.42).

### Logistic regression – Odds ratio of incorrect answer to correct answer by number of CDRs

The Generalized Estimating Equations binary logistic regression model for within-subjects repeated measures demonstrated statistically significant increased odds of an incorrect answer associated with the main effects of being a trainee (i.e., medical student or resident) (OR = 1.926; 95% CI [1.037, 3.577]; *p* = 0.038), per-item number of distinct *Cognitive Bias* themes (OR = 1.729; 95%CI [1.226, 2.437]; *p* = 0.002) and *Other Vulnerabilities* themes (OR = 2.014; 95%CI [1.280, 2.941]; *p* < 0.001), but not with *Flaws in Conceptual Understanding* themes (OR = 1.617; 95%CI [0.961, 2.720]; *p* = 0.070). This suggests that the odds of committing an error versus not committing an error in a given clinical case increases with each additional unique instance of CDRs traditionally theorized as being error-prone (i.e., cognitive biases and heuristics). The odds of committing an error versus not committing an error in a given clinical case also increases with each additional unique instance of *Other Vulnerabilities* (i.e., Marked Uncertainty and Emotional Reaction). For each additional distinct Cognitive Bias CDR and Other Vulnerability CDR present in a single case in our study sample, the findings suggest the odds of committing an error increases by a magnitude of approximately two-fold – similar to the increased risk for error conferred by being a trainee compared to being an attending physician. Each distinct instance of a coded *Flaw in Conceptual Understanding*, however, was not associated with increased odds of error that reached statistical significance.

## Discussion

We uniquely explored diagnostic error and CDRs in the context of multiple-choice questions, which, to our knowledge, has not been the subject of an empiric prospective investigation. By using a well-established TA approach for studying clinical reasoning processes combined with discrete, objective correct and incorrect answers from MCQs – a well-established means of assessing clinical reasoning - we believe that our design was well-suited for this purpose. Consistent with our hypothesis, we found that errors were associated with more verbalized CDRs. Specifically, this study demonstrates that an increase in the number of *Cognitive Bias* CDRs (the biases and heuristics traditionally described in the CDR literature) or in *Other Vulnerabilities* themes per item is associated with increased odds of committing an error - up to approximately two-fold - for a given item versus not committing an error. These findings support the idea that these heuristics and biases traditionally described in the CDR literature are more likely vulnerabilities for error than pragmatic strengths in clinical reasoning.

While our study design did not allow for a causal link of CDRs to error, our findings are consistent with views of error in complex adaptive systems where human errors in complex reasoning processes are just one part of the even more complex healthcare system. The interplay between complexity and error is often portrayed by Reason’s “Swiss Cheese model” [40] in which a greater number of “holes” increases the odds that a mistake may occur. This model demonstrates how complex systems with a few vulnerabilities, or “holes,” may be resilient enough to function, usually, without a noticeable “error.” In fact, diagnostic error may be considered an exemplar of the “Swiss cheese model” with previous research demonstrating an average of 5.9 contributing factors for each instance of diagnostic error [25]. Our findings linking CDRs to incorrect answers for MCQs align with this model and strongly suggest that the probability of a clinical reasoning error increases with more CDRs.

While CDRs themselves may contribute to error, it is also possible that CDRs are manifestations of other underlying factors (e.g., knowledge deficits) as CDRs are essentially labels that have not been explored mechanistically. Consistent with the hypothesis that knowledge is a fundamental element to reasoning errors [22], we identified several themes related to knowledge that were categorized as *Flaws in Conceptual Understanding*. Further, there were increased odds of error with each counted unique instance of *Flaws in Conceptual Understanding*; however, this was not statistically significant. In part, this lack of statistical significance may be due to the limitations of think alouds in assessing knowledge deficiencies. For instance, only verbalized utterances could be coded and participants may have simply refrained from verbalizing their understanding in the setting of knowledge deficiencies making think alouds a “specific,” but perhaps not a “sensitive,” tool for this purpose. In addition, all verbalized *Flaws in Conceptual Understanding* were coded and counted without regard for the use of that flawed knowledge in answering an item. Some of these verbalized *Flaws in Conceptual Understanding* may not have been critical to the reasoning process of the participants for a specific item (e.g., a participant verbalizes a misunderstanding of T-scores during the think aloud, but T-scores may have only been tangentially related to answering the clinical question). Further, participants with *Flaws in Conceptual Understanding* may have relied on other knowledge to solve the item (e.g., a participant misunderstands the mechanism and use of teriparatide, but knows enough about the other answer choices to “rule-out” incorrect answer choices and selects the correct answer choice). Also, it is possible that several themes outside of the *Flaws in Conceptual Understanding* category (e.g., Marked Uncertainty, Emotional Response, and Availability and Non-Availability Bias) may actually be manifestations of implicit knowledge deficits that were not explicitly verbalized. Given these limitations of think alouds, further research is needed to better understand the relationship of conceptual understanding and knowledge structures with both cognitive processes (e.g., CDRs) and with errors.

In addition to these findings, we are not aware of any studies to-date that have confirmed the presence of CDRs in real-time clinical reasoning activities; research has been retrospective [3, 4] and not well-suited to empirically demonstrating this association [21]. Prior work by Zwaan, et.al. tasked judges with evaluating clinical cases for the presence of CDRs and demonstrated hindsight bias - judges tended to identify more CDRs in cases with outcomes suggesting an error was made than in cases that did not suggest an error [7]. Our study mitigated the effects of hindsight bias by applying methods of consensus coding of the actual verbalized thoughts of participants reasoning through MCQs accompanied by transparent definitions and examples of those codes. Furthermore, two of three coders were blinded to the participant’s performance on MCQs in our work. As such, our study provides important evidence linking CDRs to errors that is not possible with other study designs. Our ability to code several well-described CDRs (i.e., cognitive biases) based on the verbalized reasoning processes of our participants additionally suggests the concept of CDRs can be extended to the reasoning that occurs in MCQ construct. Furthermore, and contrary to prior work [7], this study provides a proof-of-concept that coders can agree upon the presence or absence of CDRs through a constant-comparative approach. Importantly, we were also able to build on the existing CDR framework that is predominantly composed of specific cognitive biases by noting additional phenomena, defined in the *Flaws in Conceptual Understanding* and *“Other” Vulnerabilities* categories*,* that seemed to be entangled with traditional CDRs (i.e., cognitive biases and heuristics). For these reasons, we believe this study sets an important precedent for using MCQs to study cognitive errors in new ways and advances our understanding of clinical reasoning errors.

### Strengths and limitations

Compared to more common methods of investigating diagnostic error such as chart review, autopsy, and self-report, our unique approach of using a CDR-derived framework to explore MCQ-based “think aloud” data affords several advantages. First, with the MCQ there is a clear and objective metric of diagnostic error that limits the possibility of missing cases of error. Second, we can evaluate all cases regardless of case outcome. With the several of the more common approaches noted above, only those instances where there is a known or suspected error are studied. In this study design, we code explicit cognitive behaviors for all items allowing a comparison of cognition occurring during those instances with “error” (i.e., incorrect answer) and those without “error” (i.e., correct answer). Third, our approach allows us to increase the available sample size of “cases” to explore. This opens the possibility of researching both strengths and weaknesses in reasoning in future work. Fourth, the MCQ items in this study were developed by expert question writers and went through peer-review prior to extensive psychometric evaluation among a national sample of physicians and physicians-in-training [29, 30]. Fifth, the TA protocol used for collecting data on cognitive processes is well established in clinical reasoning research [31]. Sixth, we used a clinically-derived CDR framework established in the diagnostic errors literature. By using and building upon this framework, the findings from this work can contribute to the larger body of clinical error research. Seventh, this approach allows for a focus on the cognitive phenomena associated with error independent of the systems contributions to error. Eighth, this approach in coding somewhat insulates the results from hindsight bias by blinding coders to the correctness of the answer for each MCQ item while limiting codes to labels of specific verbalized phenomena, not judgments of reasoning quality. Overall, this approach sets a precedent for a more standardized and controlled method that could later be modified to explore this area with greater rigor as called for by *Improving Diagnosis* (2015) [3].

Limitations of our study include the small sample (14 participants) all recruited from the same academic health center. However, the performance of our study sample is consistent with the performance of a large national sample recorded by the American College Physicians. Due to the time commitment, each participant only completed 15 MCQ items with a corresponding think aloud. We also used a retrospective TA methodology. While we did this to avoid altering participants’ thinking while completing the MCQs and we carefully followed recommendations for this use of the TA, it is possible that participants’ verbalizations reflect their post hoc explanations rather their actual reasoning with answering the MCQs. The view that reasoning during clinical-vignette MCQs is similar to “native,” or “real-world,” clinical reasoning is also controversial and may be viewed as a limitation; however, there are several studies with evidence to support the similarities of reasoning processes in these different contexts [26–28]. Larger investigations may be helpful in studying the nature of the association of specific CDRs with errors and the interactions of CDRs with contextual factors (i.e., fatigue, time constraints, language barriers, electronic health records, interruptions, multi-tasking, “difficult” patients, etc.) [3, 10–15]. We performed think alouds following each block of related items (vs after each item) and performing think alouds following each item may have provided a more in depth understanding of thinking on the item level. Finally, we recommend repeating our study in more authentic practice environments (e.g., with standardized patient encounters) to determine if our findings are replicable to other settings.

## Conclusions

In summary, this study empirically links CDRs to errors and supports the view that CDRs may increase the likelihood of error for any given level of clinical experience - from attending physicians with decades of clinical experience to trainees (i.e., residents and students). Each additional unique *Cognitive Bias* CDR – those heuristics and biases classically described in the literature - demonstrated by a participant for a clinical-vignette MCQ was associated with statistically significant increased odds of error versus no error for a given MCQ. The novel approach of this study also suggested a potential mechanism for understanding, assessing, and further studying the interactions of reasoning processes and knowledge structures with errors. Given the frequency and potentially devastating consequences of error, we believe such research is critical to advance the fields of patient safety and clinical reasoning, develop new approaches to teaching clinical reasoning and bolster resilience to reasoning errors in real-world clinical practice.

## Additional file

Additional file 1: Multiple Choice Question (MCQ) Items – Content Domains, Source, Psychometrics. (DOCX 100 kb)

## Abbreviations

ABIM: American Board of Internal Medicine
ACP: American College of Physicians
CCA: Constant-Comparative Approach
CDRs: Cognitive Dispositions to Respond
IM: Internal Medicine
MCQ: Multiple-Choice Question
MKSAP: Medical Knowledge Self-Assessment Program
TA: Think aloud
