# Supplementary material for: A mixed-methods exploration of cognitive dispositions to respond and clinical reasoning errors with multiple choice questions
Source: BMC Med Educ. 2018 Nov 23;18:277. doi: 10.1186/s12909-018-1372-2 (PMC6251119; doi:10.1186/s12909-018-1372-2)
Supplement: Supplementary file 1 — Multiple Choice Question (MCQ) Items – Content Domains, Source, Psychometrics. (DOCX 100 kb) [file 12909_2018_1372_MOESM1_ESM.docx]

| **Appendix. Multiple Choice Question (MCQ) Items – Content Domains, Source, Psychometrics** | | | |
| --- | --- | --- | --- |
| **Content Domain** | **Item** | **Source** | **Item Difficulty (% correct among MKSAP* cohort)** |
| Neurocognitive Decline (NCD) | NCD.1 | MKSAP 15, Neurology #30 | Moderate (67%) |
|  | NCD.2 | MKSAP 15, Neurology #40 | Easy (84%) |
|  | NCD.3 | MKSAP 15, Neurology #51 | Moderate (65%) |
|  | NCD.4 | MKSAP 15, Neurology #63 | Moderate (57%) |
|  | NCD.5 | MKSAP for Students 4, Neurology #12 | Easy**†** (est. 80%) |
| Pulmonary Infectious Disease (ID) | ID.1 | MKSAP 15, Infectious Disease #3 | Hard (18%) |
|  | ID.2 | MKSAP 15, Infectious Disease #4 | Easy (74%) |
|  | ID.3 | MKSAP 15, Infectious Disease #36 | Moderate (63%) |
|  | ID.4 | MKSAP 15, Infectious Disease #75 | Moderate (58%) |
|  | ID.5 | MKSAP for Students 4, Infectious Disease #40 | Easy**†** (est. 80%) |
| Disorders of Bone Metabolism (DB) | DB.1 | MKSAP 15, Endocrinology #28 | Moderate (44%) |
|  | DB.2 | MKSAP 15, Endocrinology #36 | Moderate (63%) |
|  | DB.3 | MKSAP 15, Endocrinology #94 | Moderate (60%) |
|  | DB.4 | MKSAP 15, Endocrinology #110 | Easy (71%) |
|  | DB.5 | MKSAP for Students 4, Endocrinology #32 | Easy**†** (est. 80%) |

*****Medical Knowledge Self-Assessment Program = MKSAP. **†** No psychometric data were available; however, questions were taken from MKSAP for Students 4, and were considered by default to be “Easy” given their intended audience. For comparison to study sample performance, we estimated that 80% of the MKSAP cohort would have answered these items correctly.
